# Encapsulation of Oregano (*Origanum onites* L.) Essential Oil in β-Cyclodextrin (β-CD): Synthesis and Characterization of the Inclusion Complexes

**DOI:** 10.3390/bioengineering4030074

**Published:** 2017-09-09

**Authors:** Margarita Kotronia, Eleni Kavetsou, Sofia Loupassaki, Stefanos Kikionis, Stamatina Vouyiouka, Anastasia Detsi

**Affiliations:** 1 Laboratory of Organic Chemistry, School of Chemical Engineering, National Technical University of Athens, Zografou Campus, 15780 Athens, Greece; margarita.kotronia@gmail.com (M.K.); eleni29@hotmail.com (E.K.); 2 Department of Food Quality and Chemistry of Natural Products, Mediterranean Agronomic Institute of Chania (Centre International de Hautes Etudes Agronomiques Mediterraneennes), 73100 Chania, Crete, Greece; sofia@maich.gr; 3 Department of Pharmacognosy and Chemistry of Natural Products, Faculty of Pharmacy, National and Kapodistrian University of Athens, Panepistimiopolis Zografou, Athens 15771, Greece; skikionis@pharm.uoa.gr; 4 Laboratory of Polymer Technology, School of Chemical Engineering, National Technical University of Athens, Zografou Campus, 15780 Athens, Greece; mvuyiuka@central.ntua.gr

**Keywords:** oregano essential oil, encapsulation, inclusion complexes, β-cyclodextrin, release

## Abstract

The aim of the present work was to study the encapsulation of *Origanum onites* L. essential oil (oregano EO) in β-cyclodextrin (β-CD) inclusion complexes (ICs), using the co-precipitation method. The formed β-CD–oregano EO ICs were characterized by diverse methods, such as Dynamic Light Scattering (DLS), FT-IR spectroscopy, Differential Scanning Calorimetry (DSC), Thermogravimetric Analysis (TGA), Nuclear Magnetic Resonance (NMR) spectroscopy and Scanning Electron Microscopy (SEM). UV-Vis spectroscopy was used for the determination of the inclusion efficacy and the study of the encapsulated oregano EO release profile. The interactions between host (β-CD) and guest (oregano EO) in the formed ICs were proven by the FT-IR, DSC, TG and NMR analyses. The ICs, which derived from different batches, presented nanoscale size (531.8 ± 7.7 nm and 450.3 ± 11.5 nm, respectively), good size dispersion (0.308 ± 0.062 and 0.484 ± 0.029, respectively) and satisfactory stability in suspension (ζ-potential = −21.5 ± 1.2 mV and −30.7 ± 1.8 mV). Inclusion efficiency reached up to 26%, whereas the oregano EO release from the ICs followed a continuous delivery profile for up to 11 days, based on *in vitro* experiments. The formed ICs can find diverse applications, such as in the preparation of films for active packaging of food products, in personal care products for the improvement of their properties (e.g., antioxidant, antimicrobial, etc.), as well as in insect repellent products.

## 1. Introduction

Oregano is an aromatic plant which is commonly found growing wild in the countries of the Mediterranean basin [1,2]. In fact, although the name “Oregano” is used for several plants of different families and genera, the majority of the most well-known oregano plants, which have been traditionally used in cookery as food flavorings, as well as in the preservation of food products, belongs to the *Origanum* genus of the Lamiaceae or Labiatae family [1,2,3]. Although the *Origanum onites* L. plant and its essential oil (EO) are known and have been used for centuries, in the last decades the *Origanum onites* L. EO has attracted the attention and the interest of the researchers and the industry, gaining extensive popularity and, consequently, proliferating its fields of application [3]. This occurred due to the growing social awareness and demand for safer, healthier and minimally processed products that present a more close-to-natural image, as well as respect product quality and environment [1].

The *Origanum onites* L. EO has been reported to present remarkable biological properties. Particularly, it has been shown to exhibit powerful antimicrobial, antimycotic, antioxidant, anti-inflammatory and insecticidal activity, which result from its phenolic content and mainly from its major component, carvacrol [1,2,3], having also been suggested to present a significant potential in preventing neurodegenerative disorders [1,2]. Furthermore, having been granted “Generally Recognized as Safe (GRAS)” status (ESO, GRAS—182.20) [1], the *Origanum onites* L. EO can be used in products without further approval. Consequently, it fulfils the requirements of promoting well-being and being environmentally friendly, meeting the consumers’ expectations and demands [1,2,3]. Therefore, these quality characteristics render it as a valuable natural resource with great potential for industrial use, finding numerous applications among others in the food and pharmaceutical industry [1,2,3]. Certainly, the introduction and implementation of new technologies, which render the treatment of EOs and their use in several products more feasible and easier [1], have contributed to the increasing use and applications of the *Origanum onites* L. EO.

However, the EO’s hydrophobic nature, volatility and high sensitivity in the presence of light, oxygen and heat, limit its use [4,5,6,7]. Concurrently, if it is going to be embedded into a complex product matrix, it is quite possible that its sensitivity could cause considerable problems to the product, concerning the organoleptic profile, physical stability and chemical integrity [5,6,7,8]. Hence, encapsulation of EOs in biodegradable nano-delivery systems seems to be a promising, viable and efficient approach for overcoming these problems. Specifically, nano-delivery systems would permit its targeted and controlled release, as well as the increase of its aqueous solubility and stability against evaporation and exposure to light, oxygen, or heat, maintaining its quality, maintaining or even improving its properties and protecting products from undesired alterations [8,9,10,11]. Consequently, encapsulation could also be an answer to consumers’ demands for production of functional products with higher nutritional value, fewer synthetic additives and better organoleptic features [12].

Cyclodextrins (CDs) are cyclic, water-soluble oligosaccharides, which are produced from the enzymatic conversion, degradation and cyclization of starch and other related α-1,4-glucans, by CD glycosyltransferase and partly by α-amylases. They are composed of several D-glucose units linked by α-D-(1→4) linkages, exhibiting a three-dimensional structure, which is considered as a truncated cone. The most common of these ring-shaped molecules are α-CD, β-CD and γ-CD, which consist of six, seven and eight D-glucose units, respectively. Their depth is the same, irrespective of the glucose unit number, whereas their diameter, which is determined by the number of the glucose units, is different [5,6,7,11,12,13,14,15,16].

CDs have been extensively applied in various sectors as encapsulants of several organic substances, such as flavors, essential oils and spices, as well as individual compounds, such as carvacrol, thymol, menthol, ethylene and vanillin, as they present an inner hydrophobic cavity and a peripheral hydrophilic zone. Moreover, they are nontoxic, biodegradable polymers, not absorbed in the upper gastrointestinal tract, but completely metabolized by the gut microflora [5,6,7,11,12,13,14,15,16].

In particular, because of their hydrophobic cavity and hydrophilic external surface, CDs present the ability to interact with hydrophobic bioactive compounds and molecules (guest molecules), encapsulating them in their cavity. As a result, non-covalent CD—bioactive agent molecular complexes, which are also known as host—guest inclusion complexes (ICs), are formed. These complexes are mainly driven by hydrophobic or Van der Waals interactions, while their formation constitutes a dynamic equilibrium, allowing the guest molecule to diffuse reversibly from the CD cavity. Therefore, CDs constitute favorable and suitable molecules for the encapsulation of poorly soluble, temperature sensitive, or chemically labile bioactive agents, in order to protect them against diverse environmental conditions, to improve their physical and chemical stability, to retain or even enhance their biological properties, as well as to improve or extend their physical and chemical properties [5,6,7,11,12,13,14,15,16].

To our knowledge, a significantly large part of the current literature is focused on the encapsulation of the main oregano EO component, carvacrol [5,15,17,18,19], whereas little systematic research has been conducted to evaluate the encapsulation of oregano EO, with most of the studies dealing with encapsulation matrices other than β-CD [1,2,6,17,20,21,22,23] or using other methods like spray-drying [24]. In the current study, a detailed research on the encapsulation of the oregano EO in β-CD was conducted, using the co-precipitation method. The formed ICs were characterized using diverse analytical techniques, and the main parameters that affect the encapsulation procedure were investigated. In addition, the release profile of the encapsulated oregano EO in media of different temperatures and pH values was determined.

## 2. Materials and Methods

### 2.1. Materials

Oregano (*Origanum onites* L.) essential oil (EO) of food-grade quality was kindly supplied by the Laboratory of Nutritional Physiology and Feeding, of the Animal Sciences and Aquaculture Faculty, of the Agricultural University of Athens (A.U.A., Athens, Greece). β-Cyclodextrin (β-CD) of >99% purity was purchased from Fluka (Gillingham, England), while ethanol of analytical reagent grade was purchased from Merck Millipore (Billerica, MA, USA). Ethyl acetate of ACS grade was purchased from Chem-Lab (Zedelgem, Belgium). For the preparation of solutions double deionized water was used. The aforementioned materials were used without further purification.

### 2.2. Preparation of β-CD—Oregano EO Inclusion Complexes (ICs)

The co-precipitation method of Sun et al. [15], slightly modified, was used to prepare the β-CD—oregano EO ICs. Briefly, β-CD was dissolved in defined volumes of ethanol and double deionized water mixture (1:2 v/v), in order for each time a concentration of 100 mg of β-CD per 1 mL of solvents’ total volume to be achieved. In a typical experiment, β-CD (500 mg) was dissolved in ethanol-double deionized water solution (5 mL). The solution was magnetically stirred at 55 ± 2 °C, until the complete dissolution of β-CD. Subsequently, oregano EO (125 mg) was added dropwise in the β-CD solution, in order for a EO/β-CD ratio of 20:80 w/w to be obtained. The formed emulsion was continuously stirred at room temperature, until the β-CD–oregano EO ICs were formed. The elimination of the oily phase from the emulsion indicated the formation of the ICs. The final dispersion was kept in the refrigerator for approximately 1 h, after which the β-CD–oregano EO ICs were recovered by vacuum filtration, using a Hirsch filter funnel (pore size 3). The recovered ICs were washed twice with ethanol, in order for the unencapsulated oregano EO or the oregano EO absorbed on the surface of β-CD to be removed, and they were dried in vacuo for 3 h at 40 °C. Finally, 445 mg of dried ICs were obtained and stored in airtight glass containers, under refrigeration, for further analysis and characterization.

### 2.3. Characterization of the β-CD—Oregano EO ICs

#### 2.3.1. Dynamic Light Scattering (DLS)

Size, size distribution (PDI) and zeta-potential (ζ-potential) determinations were performed by Dynamic Light Scattering (DLS) method, using the Zetasizer Nano ZS device (Malvern Instruments, Malvern, UK). The samples for the DLS measurements were adequately diluted aqueous solutions of the final dried β-CD—oregano EO ICs (pH 6), prepared by dispersing 1 mg of ICs in 4 mL of double deionized water and the solution being left in stirring for 48 hours at room temperature. For both size and ζ-potential measurements, folded capillary cells DTS1070 were used, while for each sample the measurements for the size, PDI and ζ-potential were carried out at 25 ± 1 °C and in triplicate. The results were reported as mean ± standard deviation (SD).

#### 2.3.2. Fourier Transform Infrared Spectroscopy (FT-IR (ATR) Spectroscopy)

The ICs’ formation, as well as the interactions between the oregano EO and the β-CD, were confirmed and determined by Fourier Transform Infrared spectroscopy (FT-IR spectroscopy), using a JASCO FT/IR-4200 spectrometer (Japan Spectroscopic Company, Tokyo, Japan). The IR analyses were conducted in the final dried β-CD–oregano EO ICs, while for each sample, the IR analysis was carried out in the scanning range of 650–4000 cm^−1^.

#### 2.3.3. Differential Scanning Calorimetry (DSC)

The thermal characteristics of the ICs were studied with the Differential Scanning Calorimetry (DSC) method, using the DSC 1 STAR^e^ System device (Mettler Toledo, Columbus, OH, USA). DSC analyses were conducted in the final dried β-CD—oregano EO ICs, as well as in β-CD and pure oregano EO. The samples were heated from 25 °C to 400 °C, respectively, with a heating rate of 10 °C/min under nitrogen gas flow (20 mL/min).

#### 2.3.4. Thermogravimetric Analysis (TGA)

Thermogravimetric (TG) analyses were also conducted in the final dried β-CD–oregano EO ICs, β-CD and oregano EO. The TG analyses were performed in the TGA/DSC 1 STAR^e^ System Thermobalance (Mettler Toledo, Columbus, OH, USA), with the samples being heated from 25 °C to 600 °C, at a heating rate of 10 °C/min under nitrogen gas flow (10 mL/min).

#### 2.3.5. Nuclear Magnetic Resonance Spectroscopy (NMR Spectroscopy)

The ^1^H-NMR spectra of the final dried β-CD – oregano EO ICs and β-CD were recorded on a Varian 300 MHz spectrometer (Varian, Palo Alto, CA, USA).The used solvent for the preparation of the solutions was deuterium oxide (D_2_O). The coupling constants (J) are expressed in hertz (Hz) and the chemical shifts (δ) are reported in parts per million (ppm) relative to the solvent.

#### 2.3.6. Scanning Electron Microscopy (SEM)

The SEM analyses were conducted in the final dried β-CD–oregano EO ICs, using a PhenomWorld desktop scanning electron microscope (Phenom-World, Eindhoven, The Netherlands) with tungsten filament (10 kV) and a charge reduction sample holder. The size of 100 particles from each SEM image was measured, using the embedded image analysis software (Phenom Pro Suite/ParticleMetric), and the average particle size was determined.

### 2.4. Inclusion Efficiency of the β-CD—Oregano EO ICs

The inclusion efficiency (IE) represents the percentage of oregano EO encapsulated in the formed β-CD—oregano EO ICs, relative to the total initial amount of oregano EO used:

(1) $$(\%IE)=100\times \frac{mass\;of\;the\;encapsulated\;oregan\;EO\;(mg)}{initial\;oregano\;EO\;mass\;to\;be\;encapsulated\;(mg)}$$

The % IE of oregano EO in the β-CD–oregano EO ICs was determined directly, using Ultraviolet-Visible Spectroscopy (UV-Vis Spectroscopy), by quantification of the encapsulated oregano EO. The UV-Vis analyses were conducted in the final dried ICs, being performed on a JASCO double beam V-770 UV-Vis/NIR spectrophotometer (Japan Spectroscopic Company, Tokyo, Japan). For the UV-Vis analyses, 10 mg of ICs were dispersed in 10 mL of ethyl acetate, with the solution being left in stirring for 48 h at room temperature. Subsequently, the solution was filtered in vacuo and from this resulting solution, proper volumes were obtained for the performance of the analysis. The IE was quantified by measuring the absorption of the major oregano EO component, carvacrol, at 275 nm. From this absorption the mass of the encapsulated oregano EO in the ethyl acetate–oregano EO solution was calculated and used in Equation 1, in order for the IE to be determined. Furthermore, a calibration curve of the absorbance versus the concentration of the oregano EO, constructed by standard ethyl acetate−oregano EO solutions of known concentrations, was used for the quantitative determinations of oregano EO. For each sample, the UV-Vis analysis was carried out in triplicate.

### 2.5. In Vitro Release Studies of the Oregano EO from the β-CD—Oregano EO ICs

*In vitro* release experiments were performed, in order for the oregano EO release profile from the formed β-CD—oregano EO ICs to be evaluated. The release profile of the oregano EO was studied under specified conditions, in media of different temperatures and pH values. Particularly, three release experiments were conducted: (a) using deionized water as medium, at 37 °C and under neutral pH conditions, 7.4, (b) using deionized water as medium, at 50 °C and under neutral pH conditions, 7.4, as well as (c) using phosphate buffer solution as medium, at 37 °C and under slightly acidic pH conditions, 5.5. For each experiment, 100 mg of β-CD—oregano EO ICs were dispersed into 25 mL of medium and the entire system was kept at the specified temperature and pH values, being under continuous magnetic stirring (450 rpm) for approximately 10 days. At regular predetermined time intervals, 2 mL samples from the solution were taken and were analyzed by UV-Vis spectroscopy, so that the amount of the released oregano EO as a function of time to be calculated, by measuring the absorbance at 275 nm. The quantification of the released oregano EO was performed by a calibration curve. Each time a 2 mL sample was withdrawn, 2 mL of fresh medium were added at the solutions. For each experiment, two replicates were performed.

## 3. Results and Discussion

### 3.1. Characterization of the β-CD—Oregano EO ICs

#### 3.1.1. Size, Size Distribution and Zeta-Potential (ζ-Potential)

The size, size distribution and ζ-potential of the produced ICs determine to a great extent their ability to be used in certain applications. They constitute the most important parameters in the characterization of the produced ICs. The size of the ICs affects their nature and attributes, such as their surface characteristics, their physicochemical stability, the bioactive agent’s loading and targeted delivery, as well as the bioactive agent’s release profile [25,26,27,28,29]. Nevertheless, the type of application determines the range of the values that the size of the ICs should obtain; hence, there is no optimal size. For this reason, most of the times polydispersity index (PDI), which represents the size distribution of the produced ICs, presents greater interest. The PDI is a measure of the uniformity of the ICs’ sizes, constituting a significant indicator concerning the uniformity of the attributes that the ICs present (i.e., ICs of uniform sizes bring similarly loads of the bioactive agent, which leads to a uniform release of it.). As far as the ζ-potential is concerned, it constitutes an indicator of the ICs’ stability in suspension [30]. As the absolute value of the ζ-potential increases, the greater the repulsive forces between them, reducing their tendency to aggregate; therefore, the more stable the ICs become.

In the present study the obtained size, PDI and ζ-potential values for the formed β-CD–oregano EO ICs, which were derived from different batches, are presented in Table 1. As can be observed, the size and PDI values of the formed β-CD–oregano EO ICs were reproducible, presenting no significant variations. Specifically, the formed ICs exhibited a mean diameter in the range of 450.3 ± 11.5 nm–531.8 ± 7.7 nm, while their PDI values ranged from 0.308 ± 0.062 to 0.484 ± 0.029, indicating moderately uniform size dispersion. This moderate homogeneity of the ICs populations was also being perceived through their size distribution graphs (Figure S1), in which two groups of β-CD—oregano EO ICs, regarding the size, could be observed. The obtained size and PDI values could be attributed to the strong tendency of the β-CD ICs to agglomerate, as a consequence of their self-assembly in aqueous solutions [7]. This phenomenon is attributed to the absence of strong repulsive forces between the particles, as a result of the lack of significant net charge on the β-CD ICs’ surface [7]. Generally, the agglomeration does not occur uniformly, potentially creating more variability in ICs’ size [7], something that explains the obtained PDI values. Nevertheless, the obtained size values were much smaller than that found by Hill L. E. et al. (2013) [7] concerning β-CD ICs with different EOs. Furthermore, the obtained absolute ζ-potential values for the formed β-CD–oregano EO ICs were high enough to indicate the formation of stable ICs, which present a low tendency to aggregate. Particularly, the obtained ζ-potential values ranged from −21.5 ± 1.2 mV to −30.7 ± 1.8 mV, values which render the dispersion quite stable.

#### 3.1.2. FT-IR Analysis

FT-IR Spectroscopy is a commonly used method for the examination of ICs’ structure, through the determination of the interactions between the guest molecules and the carrier material [31,32,33]. The obtained FT-IR spectra are presented in Figure S2.

In the FT-IR spectrum of oregano EO (Figure S2a) the most characteristic absorptions that could be observed are present at 2961.16 cm^−1^, 1579.41 cm^−1^, 1428.03 cm^−1^, 1251.58 cm^−1^ and 938.19 cm^−1^. Specifically, the peak at 2961.16 cm^−1^ is owed to the aromatic C-H stretching vibration, while the peaks at 1579.41 cm^−1^ and 1428.03 cm^−1^ can be attributed to the C-C stretch of the aromatic ring of carvacrol, the main oregano EO component. The characteristic absorption of the C-O stretching vibration appears at 1251.58 cm^−1^, while the absorption of the ring C-H bending vibration gives a characteristic peak at 938.19 cm^−1^.

As far as the FT-IR spectrum of β-CD (Figure S2b) is concerned, the most characteristic peaks appear at 3292.35 cm^−1^ owed to the -OH stretching vibration, at 2924.63 cm^−1^ attributed to the C-H stretching vibration, at 1643.67 cm^−1^ due to the asymmetric C-H stretching of -CH_2_, as well as at 1414.33 cm^−1^ owing to the O-H bending vibration. Additionally, the absorption at 1020.75 cm^−1^ is attributed to the C-O stretching vibration of the secondary alcohol groups that are present in the β-CD molecule.

The FT-IR spectrum of the β-CD−oregano EO ICs (Figure S2c) significantly differs from the respective ones of oregano EO and β-CD. In this particular spectrum, the most characteristic absorptions appear at 2965.14 cm^−1^, 2917.71 cm^−1^, 1644.88 cm^−1^, 1436.03 cm^−1^ and 885.58 cm^−1^. Particularly, the peak at 2965.14 cm^−1^ can be attributed to the C-H stretching vibration of carvacrol, the major component of oregano EO, while the peak at 2917.71 cm^−1^ is attributed to the C-H stretching vibration of β-CD. The peak at 1644.88 cm^−1^ results from the shift of the characteristic peak that appears at 1643.67 cm^−1^ in the IR spectrum of β-CD, and consequently, is owed to the asymmetric C-H stretching of the -CH_2_. Similarly, the peak at 1436.03 cm^−1^ could possibly result from the shift of the characteristic peak that appears at 1414.33 cm^−1^ in the IR spectrum of β-CD, being attributed to the O-H bending vibration. The peak at 885.58 cm^−1^ most probably constitutes a combined result of the overlap and shift of the peak at 1020.75 cm^−1^ of the β-CD IR spectrum and the peak at 938.19 cm^−1^ of the oregano EO IR spectrum, which, along with the characteristic absorption at 1436.03 cm^−1^, prove the encapsulation of the oregano EO in the β-CD ICs, by indicating the presence of host-guest interaction.

#### 3.1.3. Thermal Analysis by Differential Scanning Calorimetry (DSC) and Thermogravimetric Analysis (TGA)

**Figure 1:**
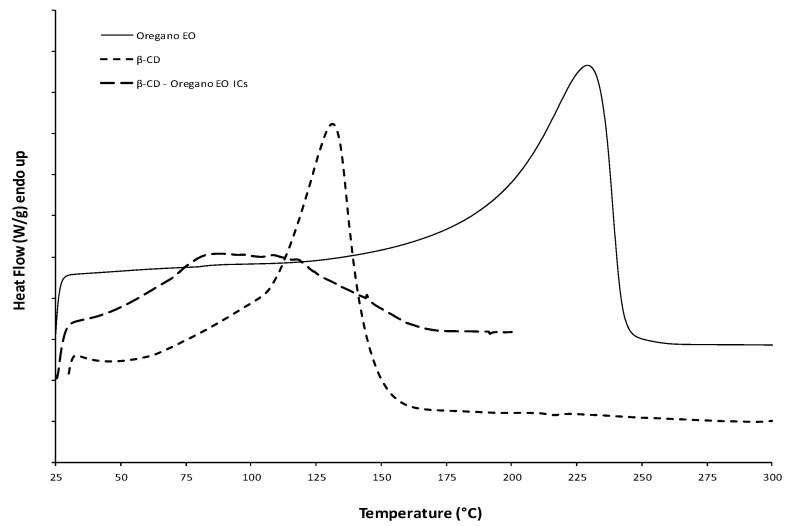
The DSC curves of the oregano EO, β-CD and β-CD—oregano EO ICs.

**Figure 2:**
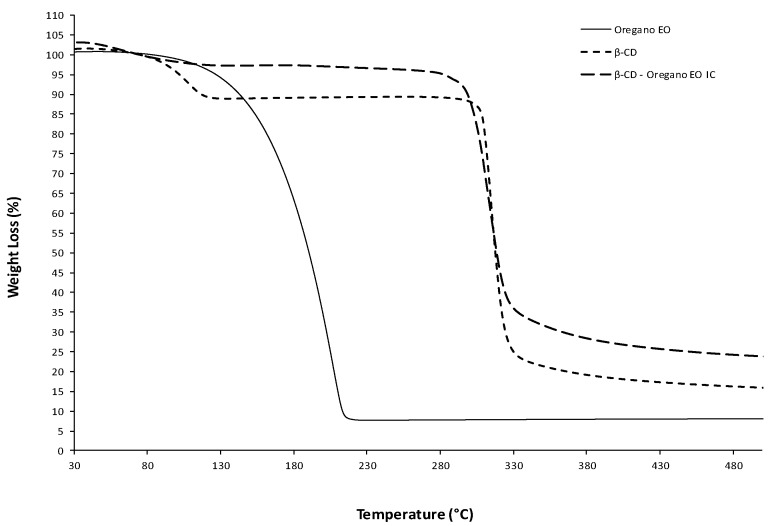
The TGA graphs of the oregano EO, β-CD and β-CD—oregano EO ICs.

Thermal analysis was conducted in order to confirm the formation of the ICs. Figure 1 presents the DSC curves under nitrogen flow of the three samples: β-CD, pure oregano EO, and β-CD−oregano EO ICs. Starting with the oregano EO, it is liquid at room temperature; therefore, it exhibited an endothermic peak at 228.2 °C that corresponds to the boiling point. The relevant thermal transition was also seen in its TGA graph (Figure 2), where the oregano EO weight loss starts at roughly 80 °C, reaching a maximum rate at 207.4 °C, with a residue at 8%. As far as the β-CD DSC curve is concerned, the observed endothermic peak at 130.9 °C can be attributed to water evaporation from the β-CD cavity, as it was suggested in the work of Karathanos et al. [34] and Gomes et al. [35] for the β-CD endotherm peaks at 175 °C and 165 °C, respectively. For the same reason of water elimination, ca. 10% weight loss is observed in the β-CD TGA graph (Figure 2) in the temperature range of 60 °C–120 °C. On the other hand, in the DSC of the β-CD–oregano EO ICs, the endothermic peak of the oregano EO disappeared and the curve was found slightly different compared to the respective one of the β-CD, indicating the ICs formation. This is in agreement with the work of Seo et al. [36], where the endothermic peak of eugenol boiling point also disappeared in the DSC curve of the relevant β-CD ICs.

The TGA results (Figure 2) also confirmed the ICs’ formation. The TGA graph of the β-CD—oregano EO ICs follows a similar pattern to the TGA graph of the β-CD, with slightly higher weight residue (27% and 18%, respectively) and maximum degradation temperature at ca. 320 °C. In particular, in the β-CD–oregano EO ICs TGA graph, the first step of weight loss in the range of 60–120 °C was not so distinctly observed as it was in the β-CD TGA graph, something that could be attributed to the partial displacement of the water molecules in the β-CD cavity by the encapsulated oregano EO. Additionally, in the range of 80–220 °C, where the oregano EO loss occurs, no significant weight loss is observed, something that suggests the protection of the oregano EO by being inside the β-CD cavity.

#### 3.1.4. Nuclear Magnetic Resonance (NMR) Analysis

**Figure 3:**
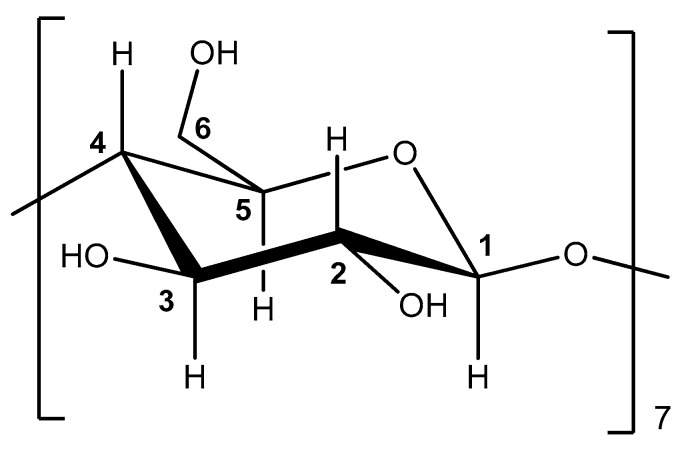
Structure of β-CD showing the numbering of β-glucose monomer.

NMR can provide useful evidence to support the formation of the ICs and help structure characterization [14,37,38]. The NMR analysis of β-CD and β-CD—oregano EO ICs was performed at 300 MHz in D_2_O. The structure of β-CD showing the numbering of β-glucose monomer is shown in Figure 3.

The ICs’ formation can lead to changes of the chemical shifts of H-3 and H-5, which are located in the interior of the β-CD cavity, and possibly the chemical shift of H-6, which is located near the cavity (Figure 3). These changes can provide information concerning the inclusion mode and binding affinity between β-CD and guest molecule. The ^1^H-NMR spectra of β-CD and β-CD–oregano EO ICs are shown in Figure S3. The Δδ values of selected NMR signals of β-CD before and after the formation of the ICs are presented in Table 2.

The H-3 and H-5 β-CD proton signals showed an upfield shift (Δδ = −0.030 ppm and −0.039 ppm, respectively) after the formation of the IC with oregano EO (Table 2). This is in accordance with the work of Locci et al. [38], who studied the inclusion of carvacrol in β-CD. A Δδ = −0.023 ppm for H-6 was also observed, whereas for the rest of the β-CD protons no noteworthy changes in their chemical shift values were detected. The significant differences in chemical shifts observed for H-3 and H-5 corroborate that an IC between the oregano EO constituents and β-CD is formed. The shift of the signal of H-5 allows also for the multiplicity of the signal (doublet) to be revealed in the spectrum of the ICs.

The observed shielding of H-3 and H-5 in the ICs can be attributed to the hydrophobic interactions with the guest molecules (oregano EO constituents), which are located inside the β-CD cavity. The downfield shift of the signal of H-6 is also indicative of an interaction between the oregano EO constituents, potentially trapped on the outer β-CD surface, with these protons which rest near the cavity of β-CD. This assumption is supported by the appearance of signals at the aromatic region (6.7–7 ppm) and the region 1–2.2 ppm, which can be attributed to some of the oregano EO constituents located on the surface of β-CD.

#### 3.1.5. Morphology

**Figure 4:**
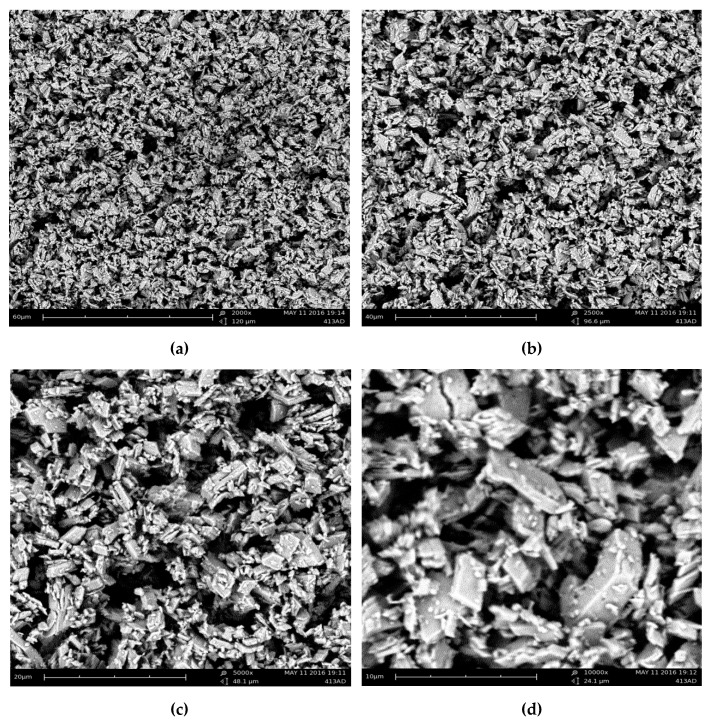
SEM images of the formed β-CD—oregano EO ICs at (**a**) × 2000, (**b**) × 2500, (**c**) × 5000 and (**d**) × 10000 magnification.

Evidence concerning the morphological characteristics of the formed β-CD—oregano EO ICs was obtained using SEM. Figure 4 presents the SEM images of the ICs at (a) ×2000, (b) ×2500, (c) ×5000 and (d) ×10,000 magnification. 

**Figure 5:**
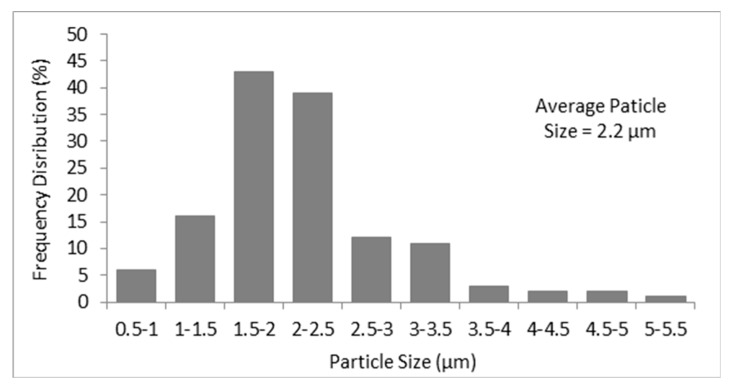
Size distribution bar chart of the particles, resulted from the analysis of the SEM images.

As can be observed, the β-CD–oregano EO ICs present a non-spherical morphology which resembles that of prisms, having parallel and rather smooth sides. Moreover, the images reveal that the β-CD – oregano EO ICs form agglomerates of different sizes, with the larger particles possibly attracting the smaller ones, while a size analysis of the particles through the obtained SEM images revealed an average particle size of 2.2 μm (Figure 5). Similar observations have been previously reported by Ikuta et al. [39], Dima et al. [40] and Rakmai et al. [31].

### 3.2. Inclusion Efficiency of the β-CD—Oregano EO ICs

Determination of the inclusion efficiency (IE) of the β-CD–oregano EO ICs is of great importance, as it provides a direct estimation concerning the effectiveness of the oregano EO encapsulation, as well as evidence concerning the available oregano EO quantity in the ICs, which along with its release profile affect directly the way that the ICs are going to be used in several applications.

The obtained IEs for the formed β-CD–oregano EO ICs that derived from different batches, as determined through Equation (1), are presented in Table 3. The IEs were significantly lower than the ones reported in the literature for the main oregano EO component, carvacrol [31,41]. This was expected as the EOs constitute complex mixtures of different compounds that present high affinities for CD molecules, competing against each other for IC formation with the β-CD [7,31]. Nevertheless, the obtained IEs were reproducible and, interestingly, much higher than the range of the theoretical maximum loading for β-CD with other EOs (8%–12%) [42], suggesting an efficient encapsulation of the oregano EO in β-CD. Moreover, the obtained IEs were in accordance with those of Parris N. et al. (2005) [20], who studied the encapsulation of EOs, including that of oregano, in zein nanospherical particles.

### 3.3. In Vitro Release Studies of the Oregano EO from the β-CD—Oregano EO ICs

The study of the oregano EO release profile from the formed β-CD–oregano EO ICs was performed in order for the effectiveness of the oregano EO encapsulation, concerning the retention, as well as the controlled and targeted release of the encapsulated oregano EO, to be estimated. Concurrently, the release profile under various conditions provides crucial information concerning the range of applications that the ICs could be used.

**Figure 6:**
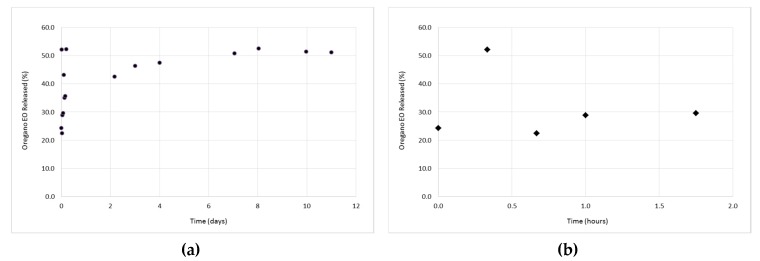
The release profile of the encapsulated oregano EO at 37 °C and pH 7.4, during 11 days (**a**), as well as at the first 1 h and 45 min of the experiment (**b**).

**Figure 7:**
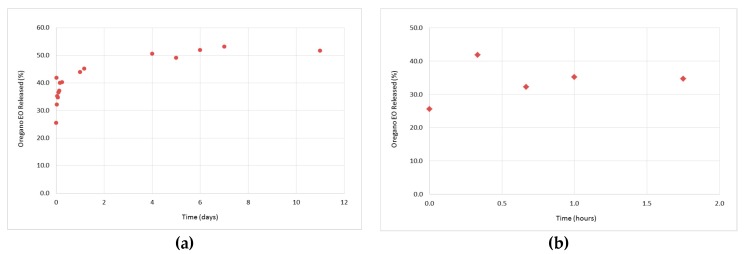
Release profile of the encapsulated oregano EO at 50 °C and pH 7.4, during 11 days (**a**), as well as at the first 1 h and 45 min of the experiment (**b**).

**Figure 8:**
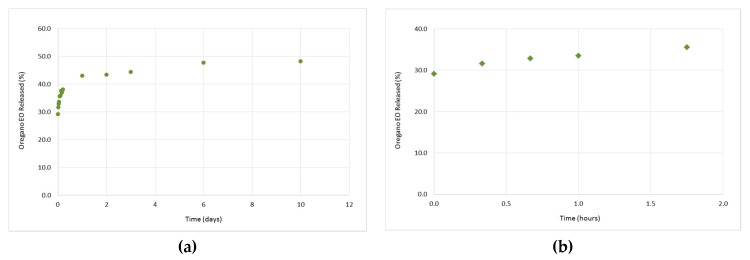
Release profile of the encapsulated oregano EO at 37 °C and pH 5.5, during 11 days (**a**), as well as at the first 1 h and 45 min of the experiment (**b)**.

The release profiles of the ICs are depicted in Figure 6, Figure 7 and Figure 8. As it can be observed in Figure 6a, at 37 °C and pH 7.4, the release profile is characterized by two different phases; an initial relatively rapid release phase (0 h–1 h and 45 min) (‘‘burst effect’’) (Figure 6b), during which a 29.6% of oregano EO was released, followed by a slower, more constant release phase (after 1 h and 45 min) (‘‘lag time’’), during which an additional 21.5% of oregano EO was released. Normally, the initial rapid release is attributed to the fraction of oregano EO which was adsorbed on the surface of the ICs, having as a result this fraction to diffuse rapidly into the medium, while at the second phase, the sustained release of oregano EO is attributed to the diffusion of the encapsulated oregano EO within the ICs cavity. After 11 days, a cumulative release of 51.2% was reached.

As far as the release profiles at 50 °C and pH 7.4 (Figure 7a), as well as at 37 °C and pH 5.5 (Figure 8a), are concerned, a similar pattern to that described above (37 °C, pH 7.4) is followed. In particular, at 50 °C and pH 7.4, a cumulative amount of 51.7% of oregano EO was released after 11 days, out of which 34.7% was released during the “burst effect” (0 h–1 h and 45 min) (Figure 7b) and 17% during the “lag time” (after 1 h and 45 min). On the other hand, at 37 °C and pH 5.5, 35.5% of the oregano EO was released within 1 h and 45 min at a fast rate (Figure 8b), while the rest 12.6% was constantly released at a slower rate for the next 10 days, after which no more oregano EO was released.

Although the oregano EO release was slightly higher at the first 1 h and 45 min of the experiments as the temperature was increased from 37 °C to 50 °C and the pH was decreased from 7.4 to 5.5 (Figure 6b, Figure 7b and Figure 8b), the release rate profiles of the formed β-CD—oregano EO ICs are very similar to each other, indicating that the changes in the temperature and pH did not significantly affect the oregano EO release. Interestingly, in all conducted experiments continuous release of the loaded oregano EO for up to 11 days was observed.

## 4. Conclusions

In the present study, the encapsulation of oregano EO into β-CD ICs was achieved. The formed β-CD–oregano EO ICs, derived from different batches, presented satisfactory characteristics concerning their size (531.8 ± 7.7 nm and 450.3 ± 11.5 nm, respectively), stability (−21.5 ± 1.2 mV and −30.7 ± 1.8 mV) and morphology, according to the obtained DLS and SEM results. The successful formation of the ICs and encapsulation of oregano EO was confirmed by FT-IR spectroscopy, NMR spectroscopy, DSC and TG analyses. The obtained results corroborate the presence of host (β-CD)–guest (oregano EO) interactions and the location of the oregano EO within the β-CD cavity, being protected inside the β-CD molecules. Moreover, the obtained inclusion efficiency was satisfactory, reaching up to 26%, while the oregano EO release profile was found to follow a continuous pattern. Hence, encapsulation of the oregano EO in β-CD could be an efficient approach to overcome the problem of its high sensitivity, as well as to enhance its bioavailability, providing the means to develop products with advantageous characteristics. The encapsulated EO can be used in diverse applications in the food, cosmetic and agriculture sectors, such as in the preparation of antimicrobial films for active packaging of food products [15,17], in personal care products for the improvement of their properties (e.g., antioxidant, antimicrobial, *etc.*) [43], as well as in insect repellent products for a safer and more environmentally friendly protection of cultivations and crops [44,45].

## Figures and Tables

**Table 1:** Size, polydispersity index (PDI) and ζ-potential average values of the formed β-CD—oregano EO ICs.

	Size (nm)	Polydispersity Index (PDI)	ζ-potential (mV)
ICs (1)	531.8 ± 7.7	0.308 ± 0.062	−21.5 ± 1.2
ICs (2)	450.3 ± 11.5	0.484 ± 0.029	−30.7 ± 1.8

**Table 2:** Chemical shift changes of ^1^H-NMR signals of β-CD and β-CD–oregano EO ICs.

Proton	Chemical Shifts (δ_1_) of β-CD Protons (ppm)	Chemical Shifts (δ_2_) of β-CD Protons in β-CD—oregano EO ICs (ppm)	Δδ = δ_2_ − δ_1_ (ppm)
Η-1	5.089	5.082	−0.007
Η-2	3.675	3.670	−0.005
Η-3	3.991	3.961	−0.030
Η-4	3.610	3.610	0
Η-5	3.875	3.836	−0.039
Η-6	3.905	3.882	−0.023

**Table 3:** Inclusion efficiency (IE) of the formed β-CD–oregano EO ICs.

	β-CD—Oregano EO ICs (*Exp. 1*)	β-CD—Oregano EO ICs (*Exp. 2*)
*Initial Oregano EO Mass (mg)*	124.60	124.70
*Encapsulated Oregano EO Mass (mg)*	28.16	31.84
*IE (%)*	22.60	25.53

## Supplementary Materials

The following are available online at www.mdpi.com/2306-5354/4/3/74/s1, Figure S1: The size distribution of the β-CD-oregano EO ICs (2); Figure S2: The IR spectra of the oregano EO (a), β-CD (b) and β-CD-oregano EO ICs (c); Figure S3: The 1H NMR spectra (300 MHz, D2O) of β-CD (a), β-CD-oregano EO ICs (expanded region 3–5.5 ppm) (b) and β-CD-oregano EO ICs (c).
