# Supplementary material for: Encapsulation of Oregano (Origanum onites L.) Essential Oil in β-Cyclodextrin (β-CD): Synthesis and Characterization of the Inclusion Complexes
Source: Bioengineering (Basel). 2017 Sep 9;4(3):74. doi: 10.3390/bioengineering4030074 (PMC5615320; doi:10.3390/bioengineering4030074)
Supplement: Supplementary file 1 [file bioengineering-04-00074-s001.pdf]

Supplementary Materials: Encapsulation of Oregano (*Origanum onites L.*) Essential Oil in β-Cyclodextrin (β-CD): Synthesis and Characterization of the Inclusion Complexes

Margarita Kotronia, Eleni Kavetsou, Sofia Loupassaki, Stefanos Kikionis, Stamatina Vouyiouka and Anastasia Detsi

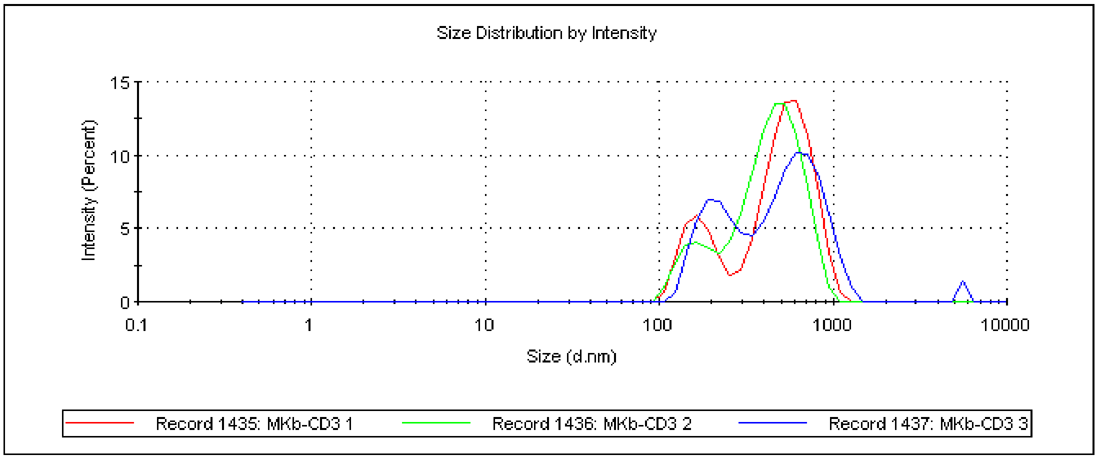

Figure S1: The size distribution of the β-CD – oregano EO ICs (2).

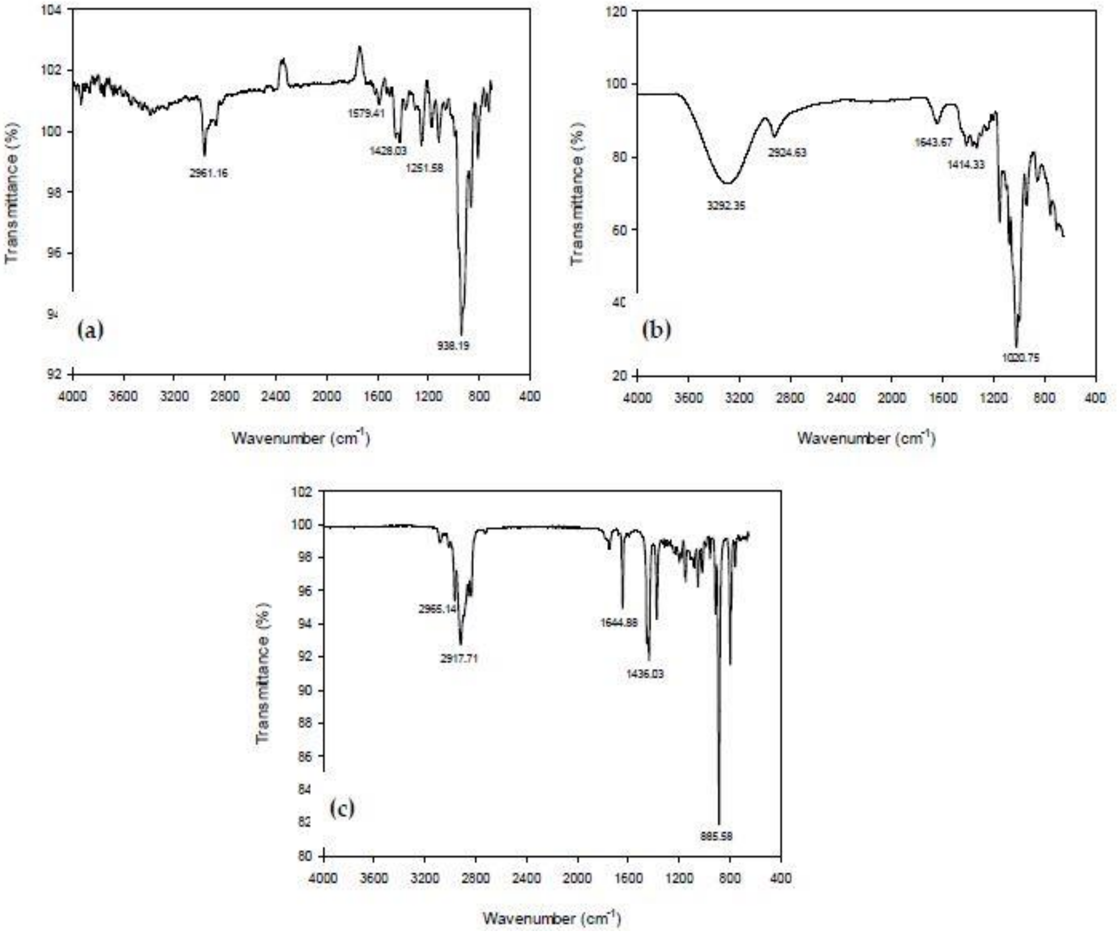

Figure S2: The IR spectra of the oregano EO (a), β-CD (b) and β-CD - oregano EO ICs (c).

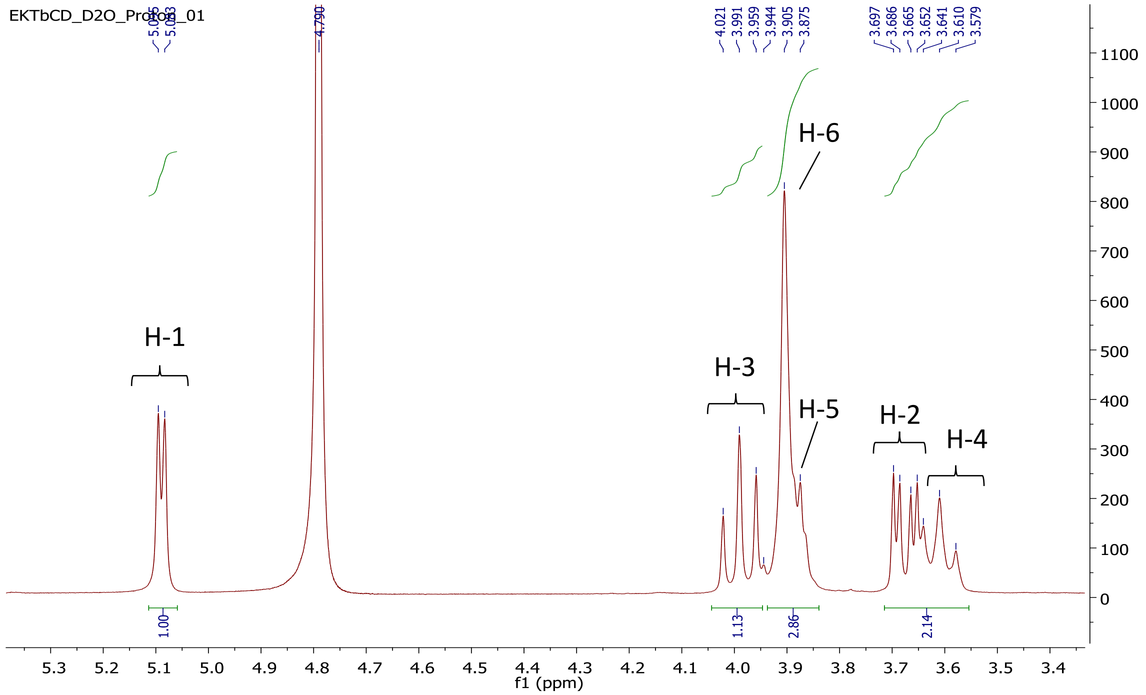
EKTbCD_D2O_Proton_01
4.790
4.021
3.991
3.959
3.944
3.905
3.875
3.697
3.686
3.665
3.652
3.641
3.610
3.579
H-1
H-3
H-6
H-5
H-2
H-4
1.00
1.13
2.86
2.14
f1 (ppm)

(a)

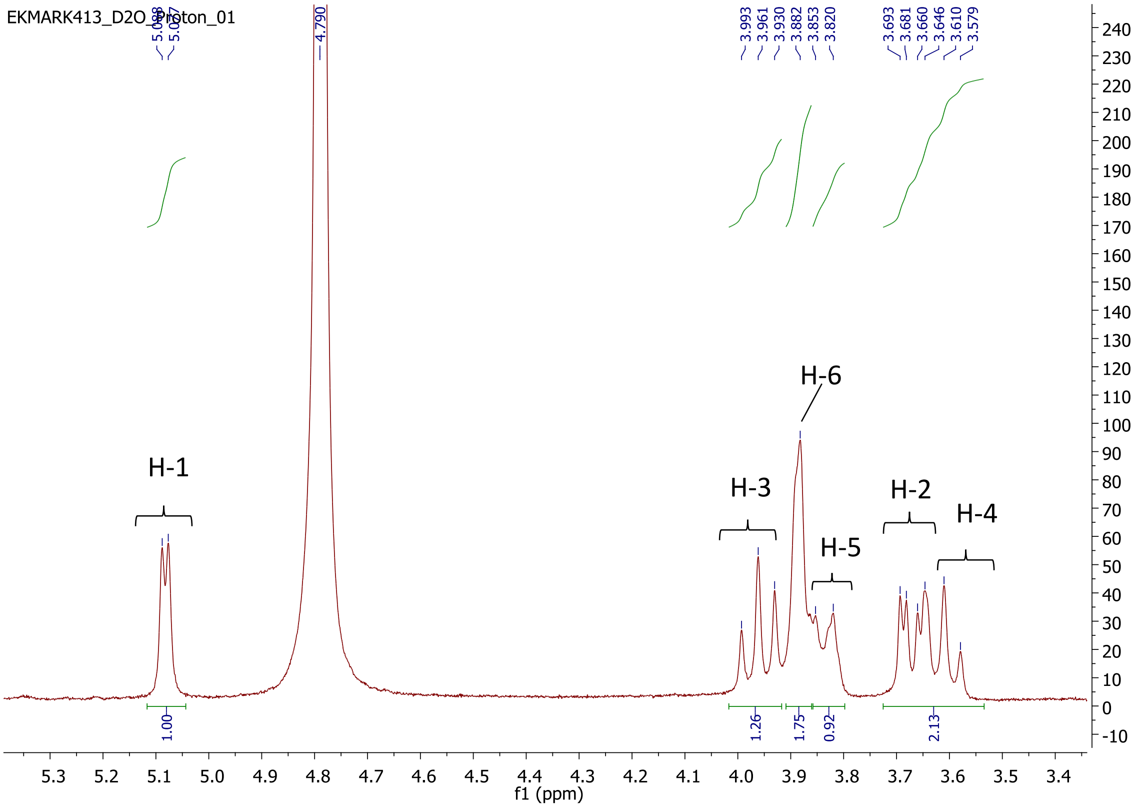
EKMARK413_D2O_Proton_01
5.098
5.087
4.790
3.993
3.961
3.930
3.882
3.853
3.820
3.693
3.681
3.660
3.646
3.610
3.579
H-1
H-3
H-6
H-5
H-2
H-4
1.00
1.26
1.75
0.92
2.13
f1 (ppm)

(b)

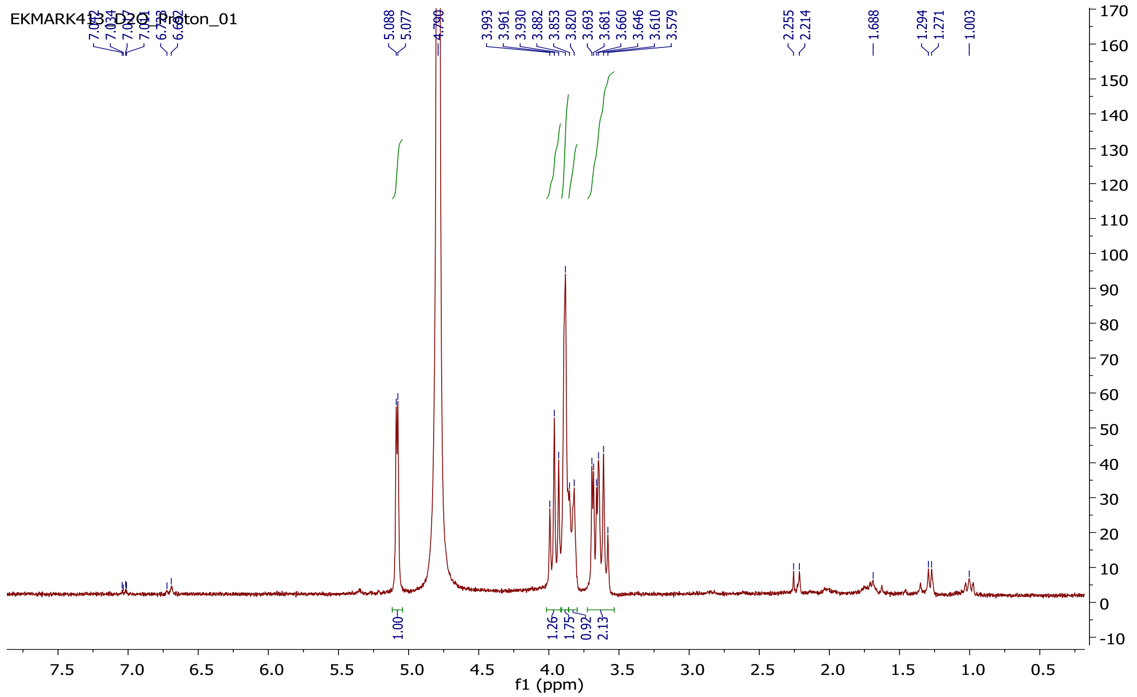

(c)

Figure S3: The ^{1}H NMR spectra (300 MHz, D_2O) of β-CD (a), β-CD – oregano EO ICs (expanded region 3 - 5.5 ppm) (b) and β-CD – oregano EO ICs (c).
